# Source-Specific PM_2.5_ Exposure and Atrial Fibrillation Risk in U.S. Older Adults: A Nationwide Cohort Study

**DOI:** 10.1021/envhealth.5c00638

**Published:** 2026-06-22

**Authors:** Qiao Zhu, Hua Hao, Yanling Deng, Yan V. Sun, Chang Liu, Alvaro Alonso, Yang Liu

**Affiliations:** † Gangarosa Department of Environmental Health, Rollins School of Public Health, 1371 Emory University, Atlanta, Georgia 30322, United States; ‡ Department of Epidemiology, Rollins School of Public Health, Emory University, Atlanta, Georgia 30322, United States; § Division of Cardiology, Department of Medicine, Emory University School of Medicine, Emory University, Atlanta, Georgia 30322, United States; ∥ Atlanta Veterans Affairs Health Care System, Decatur, Georgia 30033, United States

**Keywords:** atrial fibrillation, PM_2.5_, source apportionment, medicare, long-term exposure

## Abstract

Fine particulate matter (PM_2.5_) is associated with an increased risk of atrial fibrillation (AF), but the effects of individual PM_2.5_ sources remain unclear. We evaluated the relationship between long-term exposure to source-specific PM_2.5_ and incident AF among 26.3 million U.S. Medicare beneficiaries from 2000 to 2019. We resolved PM_2.5_ sources using the Multilinear Engine model based on PM_2.5_ components data from monitoring stations. Annual ZIP code–level exposures were assigned using inverse-distance weighting of monitoring stations within a 100 km buffer. Cox proportional hazards models were used to estimate AF risk per interquartile range (IQR) increase in source-specific PM_2.5_ concentrations, adjusting for demographic and socioeconomic factors and other environmental covariates. Over 171.6 million person-years of follow-up, 4.44 million incident AF cases were identified. Exposure to PM_2.5_ from vehicle emissions (HR 1.029, 95% CI 1.028–1.030), heavy oil and coal combustion (HR 1.048, 95% CI 1.046–1.050), secondary sulfate (HR 1.056, 95% CI 1.053–1.058), and secondary nitrate (HR 1.032, 95% CI 1.030–1.034) was significantly associated with higher AF risk. In contrast, biomass burning and soil and dust exhibited only modest associations, and secondary organic aerosols (SOA) showed no overall effect. Regional analyses revealed geographic heterogeneity: fossil-fuel–related sources drove AF risk in most regions, secondary sulfate and nitrate had the strongest effects in the southern U.S., and SOA was more strongly associated with AF in the West. These findings highlight the differential toxicity and regional heterogeneity of PM_2.5_ sources in AF risk and emphasize the need for targeted, region-specific air quality regulations focusing on traffic and industrial emissions to mitigate AF incidence in older adults.

## Graphical abstract

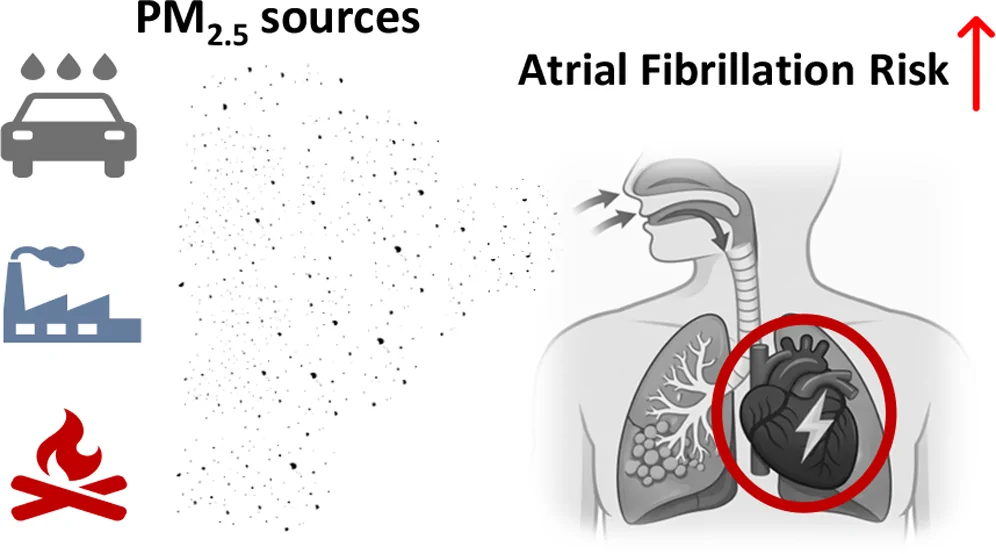

## 1 Introduction

Atrial fibrillation (AF), the most common sustained arrhythmia worldwide, affects over 33 million people and imposes a significant health burden due to its strong association with increased risks of stroke, heart failure, and premature death.^1–3^ While traditional risk factors such as advanced age, hypertension, diabetes, and cardiovascular comorbidities are well established in AF pathogenesis,^4^ growing evidence suggests that air pollution may also play an important but underrecognized role in AF development and persistence.^5^

Recent studies have increasingly shown that fine particulate matter (PM_2.5_) plays a significant role in the development of AF.^5^ Both short-term and long-term exposures to PM_2.5_ have been linked to higher AF risk. Short-term spikes in PM_2.5_ levels are associated with more emergency visits and hospitalizations, while long-term exposure has been linked to new-onset AF in otherwise healthy individuals. Meta-analyses estimate that a 10 μg/m^3^ increase in PM_2.5_ is associated with a 1–2% increase in short-term AF risk and a 7% increase in long-term risk.^6,7^ A large UK Biobank study further confirmed this association, showing a 4.4% increase in AF risk per interquartile range of long-term PM_2.5_ exposure.^8^ Furthermore, beyond its role in AF onset, PM_2.5_ may also accelerate disease progression, as elevated PM_2.5_ exposure has been linked to higher risks of stroke and all-cause mortality in individuals with existing AF.^5^ These findings underscore PM_2.5_ as an important and modifiable environmental factor contributing to both the onset and progression of AF.

Emerging evidence from cardiopulmonary and toxicological research indicates that the health impacts of PM_2.5_ are not uniform but vary substantially depending on its chemical composition and emission source.^9–12^ PM_2.5_ is a chemically complex mixture composed of both primary particles—directly emitted from sources such as traffic emissions, biomass burning, and industrial combustion—and secondary particles, such as sulfate and nitrate aerosols formed through atmospheric chemical reaction.^13^ Studies have shown that these components differ in toxicity and biological activity. For example, transition metals (e.g., iron, nickel, vanadium) commonly found in traffic and industrial emissions can catalyze the formation of reactive oxygen species, triggering oxidative stress and systemic inflammation.^12,14^ Multicity time-series and case-crossover studies have shown that cardiovascular hospitalizations are more strongly associated with PM_2.5_ enriched in metal-rich and combustion-related components than with particles from other sources.^11^ Source-apportioned studies in Atlanta further indicate that traffic-related and fossil fuel combustion sources are key drivers of adverse cardiovascular outcomes, including arrhythmia.^9,10^ Recent nationwide evidence further demonstrates that long-term exposure to source-specific PM_2.5_—particularly from oil combustion, industrial pollution, coal and biomass burning, and motor vehicle emissions—is associated with elevated atherosclerotic cardiovascular disease mortality.^15^ Despite this growing body of evidence, few studies have systematically examined the long-term effects of source-specific PM_2.5_ exposure on AF incidence in large populations. Addressing this gap is essential for identifying the most toxic contributors, understanding underlying pathophysiologic mechanisms, and guiding source-targeted air quality regulations to reduce AF burden.

In this study, we evaluated the association between long-term exposure to source-specific PM_2.5_ and incident AF among older adults in the United States, using nationwide Medicare data from 2000 to 2019 to identify incident cases In this study, we evaluated the association between long-term exposure to source-specific PM_2.5_ and incident AF among older adults in the United States, using nationwide Medicare data from 2000 to 2019 to identify incident cases. Source-specific PM_2.5_ exposures were derived from a national chemical speciation monitoring network using a receptor modeling approach, with annual concentrations assigned at the ZIP-code level. Detailed exposure assessment methods are described in the Methods section.

## 2 Materials and Methods

### 2.1 Study Population

We created a national open cohort of Medicare beneficiaries aged 65 and older to study the relationship between long-term exposure to source-specific PM_2.5_ and the risk of developing AF in the United States. To be included in the cohort, individuals had to be continuously enrolled in the medicare fee-for-service (FFS) program, have uninterrupted coverage under both medicare part A (hospital insurance) and part B (medical insurance), and live in the contiguous United States during the study period from 2000 to 2019. We censored individuals if they enrolled in a medicare advantage (HMO) plan or lost part A or B coverage at any time during follow-up, since complete medical claims data were unavailable thereafter.

We used two main data sources from the Centers for Medicare and Medicaid Services (CMS): the Medicare Denominator File and the Chronic Conditions Warehouse (CCW). The Denominator File provides yearly information on each beneficiary’s age, sex, race or ethnicity, Medicaid eligibility (as a proxy for socioeconomic status), residential ZIP code, and date of death. The CCW includes information on the presence and timing of chronic disease diagnoses, including AF, based on claims data. To reduce misclassification and ensure that only incident cases of AF were captured, we applied a three-year clean period after medicare enrollment, requiring that beneficiaries have no recorded AF diagnoses during this time. The clean period also allowed sufficient time for exposure accumulation. For example, someone who entered Medicare in 2001 would be eligible for follow-up starting in 2004 if no AF diagnosis was recorded between 2001 and 2003. This study was approved by the Emory University Institutional Review Board (IRB# STUDY00000316) and conducted under a data use agreement with CMS (DUA# RSCH-2020–55733). All data were processed and analyzed in a secure, HIPAA-compliant high-performance computing environment at the Emory Rollins School of Public Health.

### 2.2 Outcome Classification

In this study, the primary outcome was incident AF. Because the study was based on medicare administrative claims rather than primary clinical records, AF was ascertained using the CMS CCW claims-based algorithm rather than direct review of clinical diagnostic tests such as electrocardiography. The CCW algorithm identifies AF based on either one inpatient claim or two outpatient claims with relevant ICD-9 or ICD-10 diagnosis codes. The full list of ICD codes used is provided in Table S1.

### 2.3 Exposure Assessment

This study used source-specific PM_2.5_ exposure data generated from a nationwide model, ^16^which applied a multilinear engine (ME-2) receptor model to estimate long-term contributions of distinct sources to ambient PM_2.5_ concentrations across the contiguous United States from 2000 to 2019. The model utilized chemically speciated PM_2.5_ data collected from 515 monitoring stations within the EPA’s Chemical Speciation Network (CSN) and the Interagency Monitoring of Protected Visual Environments (IMPROVE) program. This data set included 21 PM_2.5_ species, comprising five major components- organic carbon (OC), elemental carbon (EC), sulfate (SO_4_^2–^), nitrate (NO_3_^–^), ammonium (NH_4_^+^)-and 16 trace elements such as bromine (Br), calcium (Ca), copper (Cu), iron (Fe), potassium (K), nickel (Ni), lead (Pb), silicon (Si), vanadium­(V), zinc (Zn), aluminum (Al), arsenic (As), chlorine (Cl), magnesium (Mg), sodium (Na), and manganese (Mn).

The ME-2 model was run separately for nine climate-based regions to better account for spatial variability in source profiles and environmental conditions. It decomposed the observed concentrations into two key matrices: source contributions (G) and species loadings (F), using both observational data and prior constraints to guide factor identification. Six major source types were consistently resolved across all climate-defined regions: soil and dust, vehicle emission, biomass burning, heavy oil and coal combustion, secondary sulfate, and secondary nitrate. Sea salt was additionally resolved in coastal regions where marine influence was more pronounced. Although secondary organic aerosols (SOA) could not be directly identified due to the absence of unique chemical markers, they were estimated indirectly based on established correlations with secondary sulfate and nitrate factors. Model performance was evaluated using multiple diagnostic approaches, including reconstruction of total PM_2.5_ mass, comparison between observed and modeled species concentrations, assessment of the stability and interpretability of source profiles, and consistency with known source tracers. Because direct ground-truth measurements for source-specific PM_2.5_ contributions are generally unavailable, these complementary diagnostics were used to evaluate the plausibility and robustness of the resolved source factors. Among these diagnostics, the reconstructed total PM_2.5_ mass was strongly correlated with the observed total mass, with correlation coefficients (*R*) ranging from 0.81 to 0.97 across regions. This metric was used as one indicator of overall model reconstruction performance and consistency between reconstructed and observed PM_2.5_ mass, rather than as the sole validation metric for source-specific estimates. For further methodological details, see Zhu et al.^16^

To match the spatial scale of available Medicare data, ZIP code–level annual source-specific PM_2.5_ exposures were estimated using an inverse-distance weighting (IDW) method. For each ZIP code, a 100 km buffer was applied to select nearby monitoring stations, and exposures were calculated as a weighted average of the source concentrations from all qualifying stations. This approach retained 72% of all ZIPs, covering approximately 77% of the total medicare study population. As Medicare residential ZIP codes may vary across years, annual estimates were linked to individuals based on their ZIP code of residence and calendar year. Because sea salt was only resolved in coastal regions and not consistently available across all geographic areas, our analysis focused on six core sources—soil and dust, vehicle emission, biomass burning, heavy oil and coal combustion, secondary sulfate, and secondary nitrate—as well as indirectly estimated SOA. The distribution of monitoring sites and ZIP code coverage are shown in Figure S1.

### 2.4 Covariates

To adjust for potential confounding in the association between long-term exposure to source-specific PM_2.5_ and incident AF, we incorporated a comprehensive set of individual- and neighborhood-level covariates. Participants with pre-existing cardiovascular conditions were not excluded from the cohort, as exclusion would substantially alter the study population and reduce generalizability. In addition, some cardiovascular conditions may lie on the causal pathway between long-term air pollution exposure and incident AF; excluding or adjusting for these conditions could introduce overadjustment bias. Demographic information—including age at Medicare enrollment, sex, race, and Medicaid dual eligibility (used as a proxy for individual-level socioeconomic status)—was obtained from the medicare denominator file.

In addition to individual-level data, we included a range of contextual variables measured at the ZIP code or county level. These included socioeconomic indicators such as population density, median household income, the percentage of elderly individuals living below the poverty line, racial and ethnic composition, homeownership rates, and education levels among older adults. To capture environmental and regional health-related factors, we also considered annual mean temperature and relative humidity, as well as county-level metrics on smoking prevalence, mean body mass index (BMI), and the availability of healthcare resources (e.g., hospital beds and physicians per capita) across five U.S. regions (Northeast, Midwest, South, Southwest, and West). Data sources included the 2000 and 2010 U.S. Census, the American Community Survey (2005–2012), the North American Regional Reanalysis (2000–2017), the Behavioral Risk Factor Surveillance System (2000–2016), and the American Hospital Association (2010, 2015, 2018). Missing values were imputed using linear interpolation or extrapolation, consistent with the approach used in prior large-scale national cohort studies.^17^ A full description of these covariates and their sources is available in detail in prior publications.^18^

### 2.5 Statistical Analysis

To investigate the association between long-term exposure to source-specific PM_2.5_ and the incidence of AF in older adults, we used stratified Cox proportional hazards models. Stratification was performed by single-year age at Medicare entry, sex, race, and Medicaid eligibility status to allow for flexible baseline hazard functions across key demographic factors. To account for spatial clustering and within-ZIP code correlation, we used a cluster-robust sandwich variance estimator at the ZIP code level. Hazard ratios (HRs) and 95% confidence intervals (CIs) were estimated per interquartile range (IQR) increase in annual average pollutant concentrations.

Covariates included in the models were informed by prior literature and covered both individual- and neighborhood-level characteristics (see Section 2.4). Models adjusted for ZIP code–level socioeconomic indicators, regional behavioral risk factors, and healthcare capacity. We also included a linear term for calendar year and categorical indicators for geographic regions to control for residual temporal and spatial trends.

To assess potential nonlinearity in the exposure–response relationship, we applied penalized spline functions to each source-specific PM_2.5_ exposure variable while adjusting for the full set of covariates. To evaluate geographic variation in the associations between source-specific PM_2.5_ exposures and AF, we tested interaction terms between each pollutant and a five-region indicator (Midwest, Northeast, Southeast, Southwest, and West) in the Cox models. We then conducted stratified analyses by region to estimate region-specific hazard ratios based on single-pollutant models, adjusting for the full set of covariates.

Several sensitivity analyses were performed to evaluate the robustness of our findings. First, we implemented a 5 year clean period—extending the original 3 year requirement described in Section 2.1—to reduce the likelihood of including participants with preclinical or undiagnosed AF at cohort entry and to minimize reverse causation. Second, to assess the influence of spatial interpolation on exposure estimates, we recalculated exposures using a 25 km buffer around each monitoring station (as opposed to the original 100 km buffer) and compared the results to evaluate potential sensitivity to the buffer size. Third, we conducted a subanalysis restricted to participants who did not change their ZIP code of residence throughout the follow-up period (i.e., the nonmover cohort) to account for potential measurement error related to residential mobility and ensure the consistency of exposure assignments over time.

## 3 Results

### 3.1 Study Population Characteristics

Table 1 summarizes the characteristics of the study population after applying a 3 year clean period to exclude individuals with potential pre-existing diagnoses of AF. The final cohort included 26,316,593 Medicare beneficiaries aged 65 years or older, contributing 171,555,756 person-years of follow-up between 2000 and 2019. The study included 26,316,593 participants, with 4,443,632 (16.9%) developing AF. At cohort entry, the mean age was 73.9 years (SD, 6.7), with 64.7% aged under 75. The median follow-up was 6 years. Females comprised a higher proportion of the cohort than males, accounting for 59.1% of the total population. Racial composition was predominantly White (86.7%), with Black beneficiaries representing 8.2%, and 5.1% identifying as other racial or ethnic groups, including Asian, Hispanic, American Indian or Alaskan Native, and unknown. Regarding socioeconomic status, 17.3% of participants were eligible for Medicaid, while the remaining 82.7% were ineligible, indicating most nondual eligible individuals in the cohort. Figure 1 shows the geographic distribution of initial AF cases per 100,000 Medicare beneficiaries across the contiguous U.S. from 2000 to 2019. Incident AF rates were notably higher in the Midwest and East, and lower in much of the Western region.

**Table 1:** Baseline Characteristics of the Study Population and Distribution of Source-specific PM_2.5_ Exposures after the 3 Year Clean Period^c^

characteristics	*N* = 26,316,593
number of AF events	4,443,632 (16.9)
total person-years	171,555,756
median follow-up years	6 (7)
age at entry (years)	73.92 (6.70)
age < 75 years	17,030,195 (64.7)
age ≥ 75 years	9,286,398 (35.3)
Sex	
male	10,757,346 (40.9)
female	15,559,247 (59.1)
Race	
white	22,817,329 (86.7)
black	2,152,267 (8.2)
other^a^	1,346,997 (5.1)
Medicaid Eligibility	
ineligible	21,761,356 (82.7)
ever eligible	4,555,237 (17.3)
PM_2.5_ Sources (μg/m^3^)^b^	
soil and dust	0.29 (0.18)
vehicle emission	1.47 (0.59)
biomass burning	1.20 (0.49)
secondary sulfate	3.27 (1.62)
secondary nitrate	1.58 (1.04)
heavy oil and coal combustion	1.11 (0.57)
SOA	0.94 (0.55)

a Other included Asian, Hispanic, American Indian, or Alaskan Native, and unknown.

b Presented as median concentration (IQR).

c Data are summarized as mean (standard deviation) for continuous variables and count (percentage) for categorical variables, unless otherwise specified. Abbreviations: SD, standard deviation; IQR: interquartile range.

**Figure 1:**
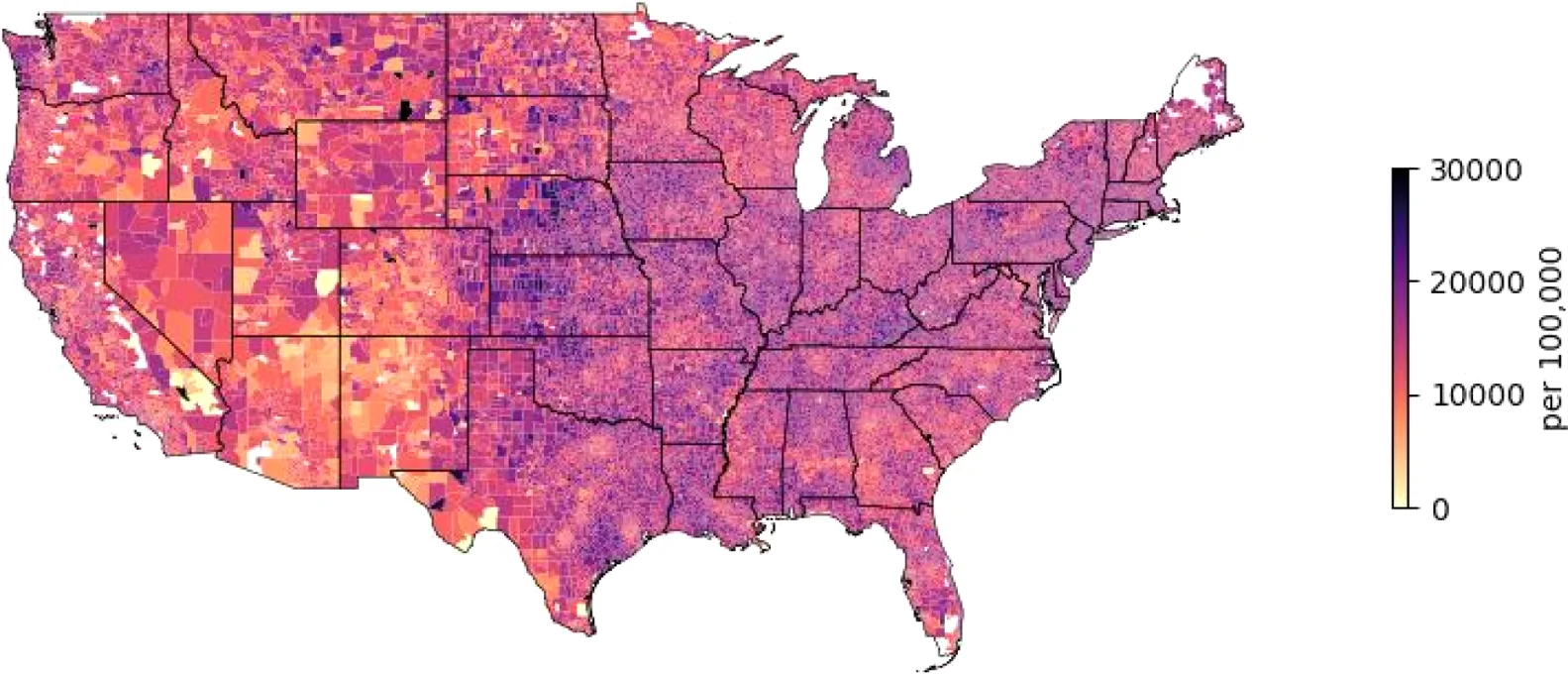
Number of incident AF per 100,000 medicare beneficiaries throughout the contiguous United States from 2000 to 2019, considering a 3 year clean period.

### 3.2 PM_2.5_ Source Exposure

Source-specific PM_2.5_ exposures were assigned at the ZIP code level using spatial interpolation based on data from national monitoring networks. To maintain geographic compatibility with Medicare records, exposure estimates were restricted to ZIP codes located within 100 km of at least one monitoring station. Among these ZIP codes, median annual concentrations varied by source: secondary sulfate showed the highest median concentration (3.27 μg/m^3^), followed by secondary nitrate (1.58 μg/m^3^), vehicle emissions (1.47 μg/m^3^), biomass burning (1.20 μg/m^3^), heavy oil and coal combustion (1.11 μg/m^3^), SOA (0.94 μg/m^3^), and soil and dust (0.29 μg/m^3^).

Figure 2 illustrates the spatial distribution of average source-specific PM_2.5_ concentrations across the contiguous United States from 2000 to 2019 for the seven nationally distributed sources examined in this study. Soil and dust concentrations were highest in the arid Southwest, particularly in desert regions such as Arizona and New Mexico. Vehicle emissions and secondary nitrate were elevated in urban and industrialized areas, especially across the Midwest, Northeast and California, reflecting high traffic density and precursor emissions. Biomass burning showed prominent concentrations in the Northwest, West, and Southeast, influenced by wildfire activity, residential wood combustion, and biomass energy production. Heavy oil and coal combustion was concentrated in the Ohio Valley and parts of the Southeast, aligning with regions historically reliant on coal-fired power generation. Secondary sulfate was most abundant in the Eastern U.S., particularly in areas affected by sulfur dioxide emissions from fossil fuel combustion. SOA levels were highest in the Southeast and California, likely due to photochemical reactions involving biogenic and anthropogenic precursors in warm, humid environments.

**Figure 2:**
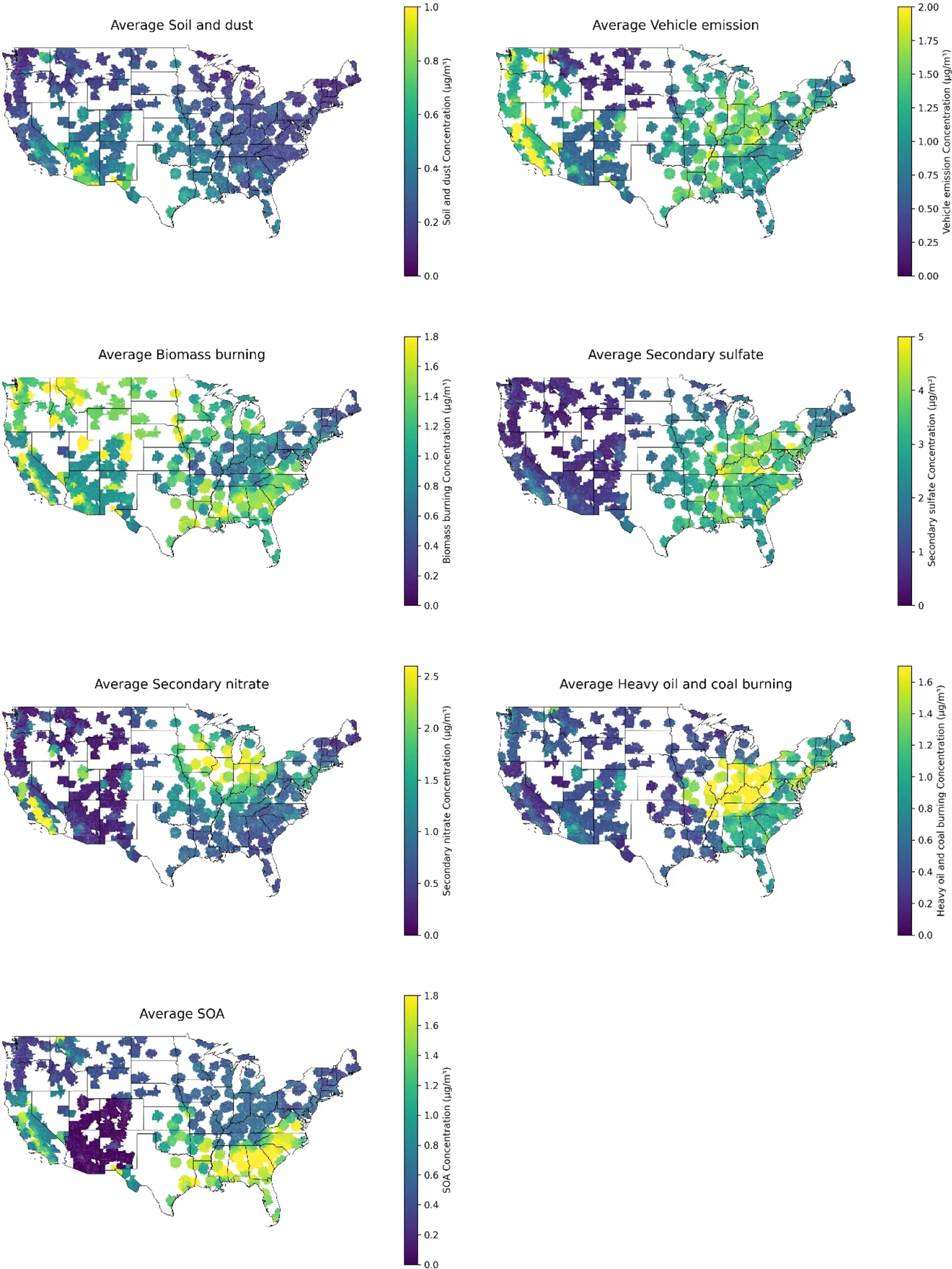
Spatial distribution of average PM_2.5_ source contributions (μg/m^3^) derived from ME-2 analysis across the contiguous United States from 2000 to 2019. Source estimates were assigned using inverse distance weighting and restricted to ZIP codes with centroids located within 100 km of a monitoring site.

### 3.3 Health Effect Estimates

Table 2 presents the associations between long-term exposure to source-specific PM_2.5_ and incident AF, estimated using single-pollutant Cox proportional hazards models. All models were adjusted for individual-level characteristics, neighborhood-level socioeconomic indicators, behavioral risk factors, and health care capacity, while also accounting for temporal and spatial trends. HRs and 95% CIs were reported for each IQR increase in exposure, as well as across quartiles of ZIP code–level concentrations.

**Table 2:** Associations between Source-Specific PM_2.5_ Exposures and AF^a^

exposure	continuous (per IQR) HR (95% CI)	quartile 1	quartile 2 HR (95% CI)	quartile 3 HR (95% CI)	quartile 4 HR (95% CI)
soil	1.006 (1.005, 1.008)	ref	1.034 (1.029, 1.039)	1.037 (1.032, 1.043)	1.035 (1.029, 1.042)
vehicle emission	1.029 (1.028, 1.03)	ref	1.042 (1.038, 1.047)	1.079 (1.073, 1.086)	1.086 (1.079, 1.093)
biomass burning	1.002 (1.001, 1.004)	ref	1.028 (1.023, 1.033)	1.005 (0.999, 1.011)	1.005 (0.999, 1.012)
secondary sulfate	1.056 (1.053, 1.058)	ref	1.039 (1.033, 1.044)	1.064 (1.057, 1.071)	1.099 (1.09, 1.108)
secondary nitrate	1.032 (1.03, 1.034)	ref	1.045 (1.04, 1.049)	1.085 (1.078, 1.091)	1.094 (1.085, 1.103)
heavy oil and coal combustion	1.048 (1.046, 1.05)	ref	0.986 (0.981, 0.991)	1.011 (1.005, 1.017)	1.058 (1.05, 1.066)
SOA	0.985 (0.983, 0.987)	ref	1.061 (1.056, 1.067)	1.035 (1.03, 1.041)	1.009 (1.003, 1.016)

a Note: models adjusted for temperature, relative humidity, smoking rate, BMI, hospital availability, and ZIP code–level socioeconomic covariates (population density, racial composition, median household income, renting rate, poverty, and education level), in addition to calendar year and geographic region of the United States.

Among the seven sources examined, secondary sulfate showed the strongest association with AF, with an HR of 1.056 (95% CI: 1.053, 1.058) per IQR increase, and a clear monotonic increase across quartiles, reaching an HR of 1.099 (95% CI: 1.090, 1.108) in the highest quartile. Similarly, secondary nitrate exposure was associated with increased AF risk (HR per IQR = 1.032; 95% CI: 1.030, 1.034), with quartile-specific HRs increasing from 1.045 in Q2 to 1.094 in Q4. Vehicle emissions also demonstrated a strong and consistent association, with an HR of 1.029 (95% CI: 1.028, 1.030) per IQR and 1.086 (95% CI: 1.079, 1.093) in Q4. Heavy oil and coal combustion showed a moderate positive association overall (HR per IQR = 1.048; 95% CI: 1.046, 1.050). Biomass burning and soil and dust exposures were more weakly associated with AF risk (HRs per IQR = 1.002 and 1.006, respectively). In contrast, SOA exhibited a negative association in the continuous analysis (HR per IQR = 0.985; 95% CI: 0.983, 0.987), although the quartile analysis revealed elevated risks at lower quartiles and a diminishing effect on higher exposures, suggesting a nonlinear relationship.

Figure 3 illustrates the concentration–response (C–R) relationships between long-term exposure to source-specific PM_2.5_ and the risk of incident AF, derived from penalized spline functions in single-pollutant Cox models. Soil and dust, vehicle emissions, and secondary sulfate all demonstrated consistent upward trends across the exposure range, with HRs increasing steadily with higher concentrations. Secondary sulfate showed the steepest slope, suggesting a strong and approximately linear risk increase, especially at lower-to-moderate exposure levels. Biomass burning and secondary nitrate also exhibited increasing trends in their most observed concentration ranges. In contrast, heavy oil and coal combustion displayed a nonlinear, bimodal pattern. The HR curve remained relatively flat at low-to-moderate concentrations but began to rise sharply beyond a threshold. Finally, the C–R curve for SOA initially increased with exposure, reaching a peak at moderate concentrations, followed by a gradual decline at higher levels.

**Figure 3:**
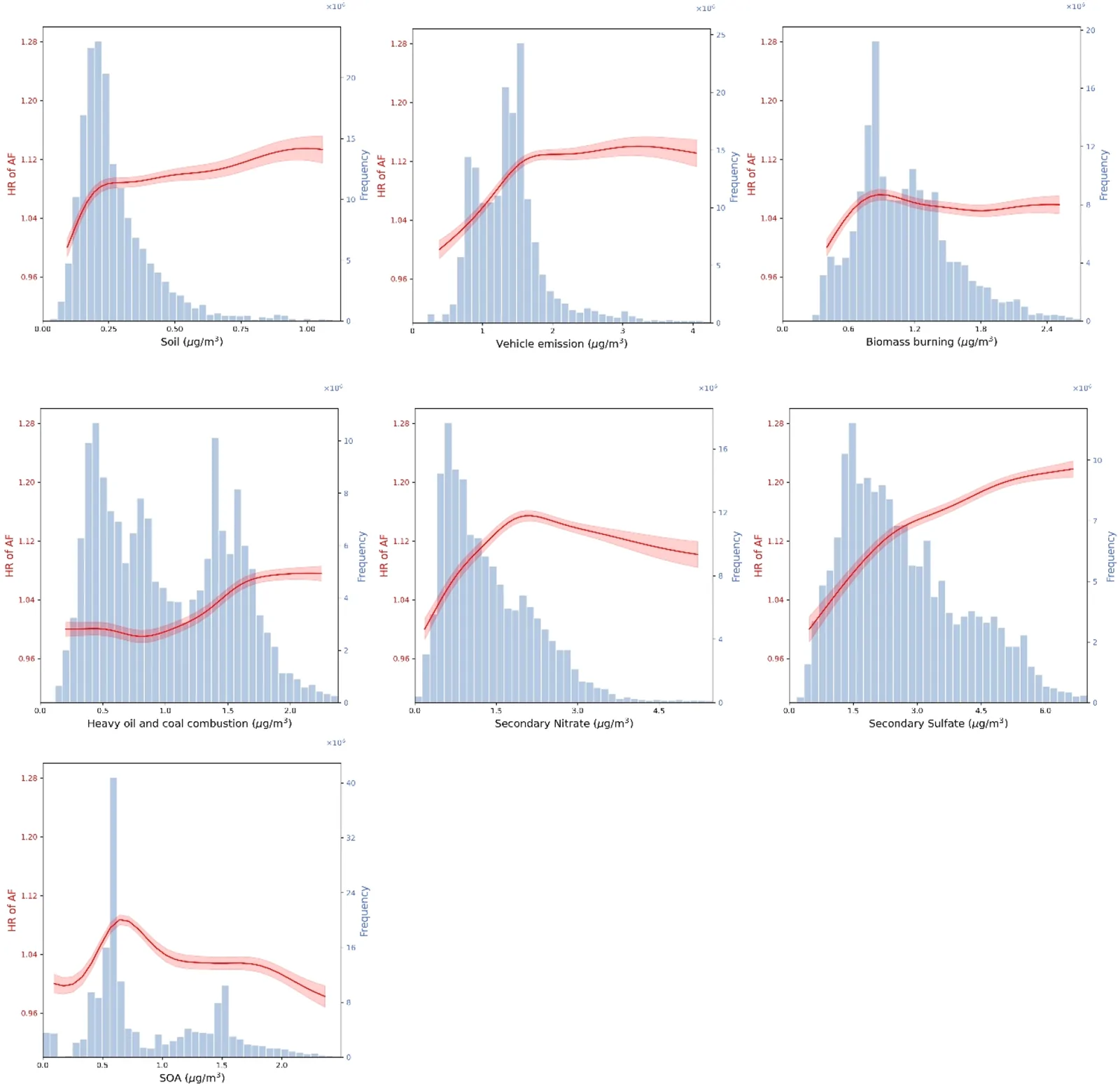
Concentration–response curves for source-specific PM_2.5_ exposures and incident AF based on single-pollutant models. Curves are shown across the 0.05th to 99.95th percentile of exposure concentrations. The reference value (HR = 1) corresponds to the 0.05th percentile of the exposure distribution. Models adjusted for temperature, relative humidity, smoking rate, BMI, hospital availability, and ZIP code–level socioeconomic covariates (population density, racial composition, median household income, renting rate, poverty, and education level), in addition to calendar year and geographic region of the United States.

### 3.4 Regional Analyses

To assess whether the associations between source-specific PM_2.5_ components and AF varied across regions, we tested interaction terms between each pollution source and the five-region indicator in the Cox models. All interaction terms were statistically significant (*p* < 0.001), providing strong evidence of regional heterogeneity in the effects of PM_2.5_ sources on AF risk.

Figure 4 presents the regional variation in the associations between source-specific PM_2.5_ exposures and incident AF, based on single-pollutant Cox proportional hazards models stratified by five major U.S. regions: Midwest, Northeast, Southeast, Southwest, and West.

**Figure 4:**
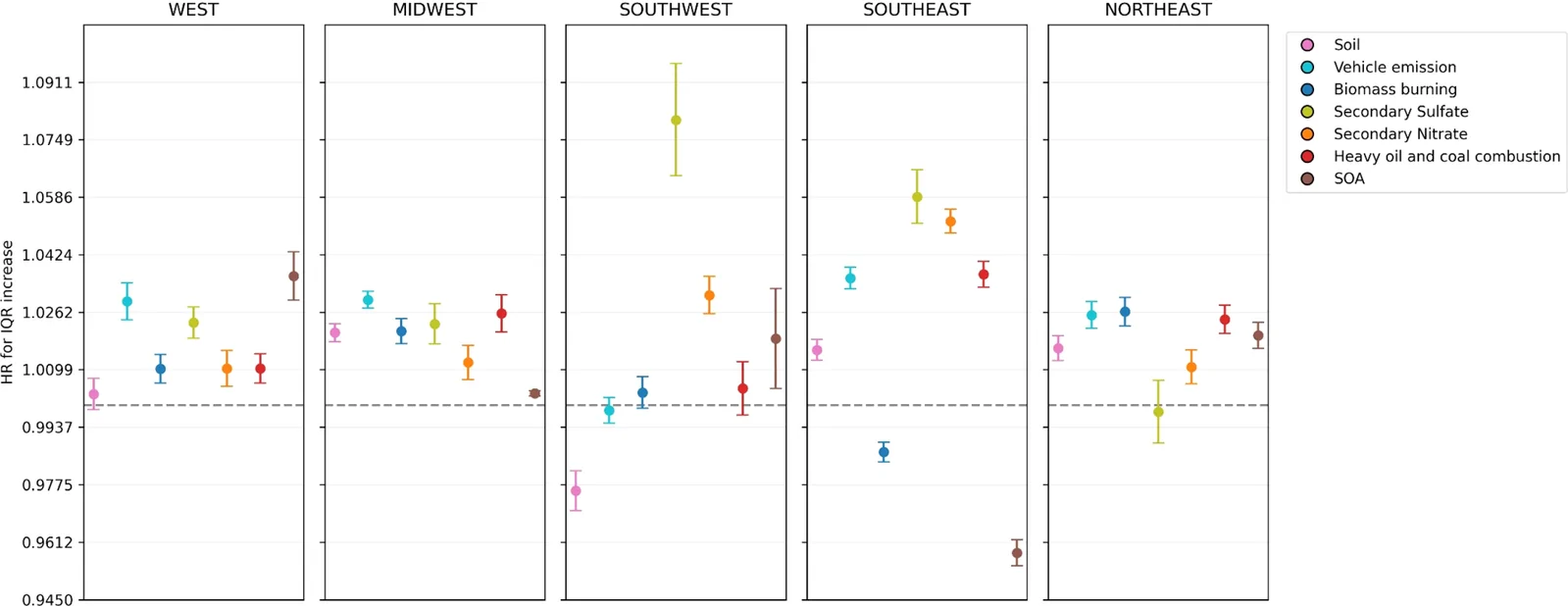
Hazard ratios (HR) of incident AF per interquartile range (IQR) increase in each source-specific PM_2.5_ exposure, stratified by U.S. region. Estimates were derived from single-pollutant Cox proportional hazards models, with error bars indicating 95% confidence intervals (CIs).

In the West, all seven PM_2.5_ sources exhibited HRs greater than 1.0, indicating consistent positive associations with AF risk. The most pronounced effects were observed for vehicle emissions and SOA. A similar pattern was found in the Midwest, where all sources were also positively associated with AF. In this region, the strongest associations were seen for vehicle emissions and heavy oil and coal combustion, suggesting the prominent role of fossil fuel combustion sources. In the Southwest, secondary sulfate and SOA showed the highest HRs among the source categories, while in the Southeast, the associations were strongest for secondary sulfate and secondary nitrate, reflecting the influence of power plant and industrial emissions in these regions. In the Northeast, vehicle emissions and biomass burning were the dominant contributors, with elevated HRs indicating their regional relevance.

In summary, these findings highlight important geographic variation in PM_2.5_ source impacts on AF. Fossil fuel–related emissions, particularly from vehicles and coal/oil combustion, were significantly associated with AF across most regions. Biomass burning was positively linked to AF in nearly all areas. Notably, secondary sulfate and nitrate had more pronounced impacts in the southern regions, while SOA was more strongly associated with AF in the West.

### 3.5 Sensitivity Analyses

The associations between source-specific PM_2.5_ exposures and AF were generally stable across several sensitivity analyses.

First, when we applied a stricter five-year clean period to define the AF-free baseline cohort (Tables S2 and S3), requiring participants to be free of AF diagnosis for five years after Medicare enrollment before follow-up, the associations for most PM_2.5_ components remained consistent with those from the three-year clean period. For biomass burning, the hazard ratio shifted from a slight positive association (HR = 1.002, 95% CI: 1.001, 1.004) to just below the null (HR = 0.997, 95% CI: 0.995, 0.999).

Second, we evaluated the influence of spatial resolution in exposure assignment by recalculating exposures using a 25 km buffer around monitoring stations, compared to the original 100 km buffer (Table S4). The associations for most PM_2.5_ components were robust to this change. Biomass burning again showed the greatest sensitivity, with the hazard ratio shifting from a slight positive association (HR = 1.002, 95% CI: 1.001, 1.004) to an inverse association (HR = 0.983, 95% CI: 0.980, 0.986). Given that biomass burning was the only source for which the direction of association changed under the alternative buffer size—and that other sources consistently showed stronger and more stable HRs—these findings suggest weaker evidence for a robust association between biomass burning and AF.

Third, restricting the cohort to nonmovers—participants who did not change ZIP codes throughout follow-up—yielded consistent results (Table S4). The HRs remained comparable or were slightly elevated relative to the main model, further supporting the stability of our findings.

## 4 Discussion

In a nationwide Medicare cohort of 26.3 million adults aged ≥65 years (2000–2019), we assessed incident AF in relation to source-specific PM_2.5_ exposure—to our knowledge, the first nationwide study to specifically evaluate the relationship between PM_2.5_ sources and AF. Long-term exposure to PM_2.5_ from fossil-fuel combustion—including vehicle emissions and heavy-oil or coal combustion—was linked to higher AF risk, as were particles from biomass burning, soil and dust, and secondary inorganic aerosols (sulfate + nitrate). By contrast, SOA showed no association with AF. These findings align with earlier reports on cardiovascular morbidity but extend them to AF specifically. Among primary sources for PM_2.5_, vehicle emissions and heavy-oil and coal combustion showed the strongest and consistently rising concentration–response curves for AF. Mechanistic evidence from epidemiological, clinical, and experimental studies supports these source-specific associations. Fossil-fuel-derived PM_2.5_, particularly that enriched in transition metals, can trigger systemic inflammation and oxidative stress, promoting atherosclerosis progression, impairing endothelial function, and directly modulating cardiac ion channels.^19–22^ These processes contribute to autonomic imbalance, myocardial ischemia, and repolarization abnormalities, which shorten atrial effective refractory periods, augment spatial electrophysiological heterogeneity, and facilitate early or delayed afterdepolarizations.^23,24^ In addition, ultrafine particles contained in fossil-fuel combustion–derived PM_2.5_ may translocate into the circulation, increasing intracellular reactive oxygen species and directly altering sodium, calcium, and potassium currents, thereby providing both triggers and substrates for AF initiation and maintenance.^25,26^ Structural remodeling, including fibrosis and chamber dilation, further slows conduction and stabilizes reentrant circuits, creating a self-perpetuating “AF-begets-AF” cycle.^25,26^ Consistent with prior literature, including a study in Dhaka, Bangladesh, fossil-fuel-derived PM_2.5_ showed stronger associations with cardiovascular morbidity and mortality than other combustion sources, including biomass burning.^27^ Additionally, the Soil and Dust source showed a positive but relatively modest association with AF (HR = 1.006). This pattern is consistent with findings from Tao et al. (2023),^28^ who applied principal component pursuits to New York City data and reported weak but positive associations between crustal dust PM_2.5_ and myocardial infarction hospitalizations. This suggests that while noncombustion sources contribute less strongly to cardiovascular risk than fossil-fuel combustion, they are not entirely innocuous.

To further examine whether the associations between source-specific PM_2.5_ and AF varied geographically, we conducted regional analyses using five U.S. Census regions: Midwest, Northeast, Southeast, Southwest, and West. The associations varied substantially across regions. For the West and Midwest, all PM_2.5_ sources were positively associated with AF, with vehicle emissions showing the pronounced effects. In the southern regions (Southwest and Southeast), secondary sulfate had the strongest impact, which primarily originates from power plant emissions.^29^ In the Northeast, the effects of combustion-related sources were consistently significant, including vehicle emissions, biomass burning, and heavy oil and coal combustion. Notably, although SOA showed no overall association with AF in the primary analysis, the regional analysis revealed that its effects were heterogeneous: HRs fell below 1 only in the Southeast, while in the West, SOA showed the strongest positive association with AF. These inverse or null associations should be interpreted with caution and do not necessarily indicate protective effects, but may reflect nonlinear exposure–response relationships, regional differences in chemical composition, or residual confounding. This regional contrast likely reflects differences in SOA precursors and formation processes. Western SOA, driven largely by wildfires, contains highly oxidized and reactive organic particles that may trigger acute cardiovascular responses.^30–32^ In contrast, Southeastern SOA is mainly isoprene-derived and forms under humid, acidic conditions, making it less acutely cardiotoxic and consistent with the null AF associations observed in our study.^33–35^ Supporting this, Pennington et al. (2019) found that SOA in Atlanta was not associated with acute cardiovascular events.^9^

This study has several strengths. First, it is based on a nationwide Medicare cohort of 26.3 million older adults followed for nearly 20 years, providing exceptional statistical power and broad relevance to the U.S. elderly population. Second, source-specific PM_2.5_ exposures were estimated from chemically resolved monitoring data through receptor modeling, enabling us to distinguish contributions from traffic emissions, fossil-fuel combustion, biomass burning, and secondary aerosol formation with greater precision than total PM_2_._5_. Third, our analysis incorporated a three-year “clean” period and included only participants with continuous enrollment in medicare fee-for-service part A and part B, ensuring the identification of newly diagnosed AF cases and providing a more precise estimate of incidence; in addition, the availability of rich individual- and neighborhood-level covariates helped minimize residual confounding from sociodemographic, behavioral, and environmental factors. Fourth, by examining geographic variation across five U.S. Census regions, the study revealed clear heterogeneity in source-specific effects, providing new insights into how regional differences in PM_2.5_ sources and composition may influence AF risk. Finally, multiple sensitivity analyses produced consistent results, supporting the robustness of our conclusions.

Several limitations should be acknowledged. Our cohort was constructed using medicare administrative data, which lacked detailed individual-level behavioral risk factors such as smoking history, alcohol consumption, and physical activity—factors known to be associated with AF risk. Although we incorporated proxy variables (e.g., county-level smoking prevalence and BMI), residual confounding from these unmeasured lifestyle factors may still exist, and such confounding is likely to be nondifferential. Our source apportionment successfully identified the major PM_2.5_ source categories but could not fully resolve highly correlated emissions. For instance, heavy oil and coal combustion were represented as a single combined factor. Exposure assessment relied on ZIP-code–level estimates, which smooth fine-scale spatial variability and cannot reflect individual differences in mobility, commuting behavior, or time spent indoors versus outdoors. Such spatial aggregation may introduce primarily nondifferential exposure misclassification, which is likely to bias the estimated associations toward the null rather than inflate effect estimates.^36^ Although we adjusted for extensive demographic, socioeconomic, and environmental covariates, residual confounding is possible from unmeasured factors such as detailed smoking history, BMI, and indoor pollutant sources. In addition, several source-specific PM_2.5_ components showed moderate-to-high correlations (Table S5). We therefore focused on single-pollutant models, as including all source-specific PM_2.5_ components and copollutants simultaneously may introduce multicollinearity and unstable estimates, complicating interpretation of source-specific effects. While meteorological factors such as temperature and relative humidity were adjusted for in the models, copollutants such as O_3_ and NO_2_ were not explicitly included; therefore, residual confounding by these factors cannot be fully ruled out. Finally, AF outcomes were identified through administrative claims, which may not capture every case; however, prior studies indicate that any resulting misclassification is likely modest and would bias results toward the null.^18^

## 5 Conclusion

In conclusion, this nationwide study demonstrates that long-term exposure to source-specific PM_2.5_ is differentially associated with AF risk among older U.S. adults. Fossil-fuel combustion sources—particularly vehicle emissions and heavy-oil and coal burning merged as the most consistent and strongest contributors, while biomass burning and soil and dust showed weaker but positive associations. Secondary inorganic aerosols were associated with AF overall, with secondary sulfate showing the most pronounced effects in the southern U.S., whereas SOA displayed no overall effect but revealed pronounced regional heterogeneity. These findings imply the importance of considering PM_2.5_ sources and geographic context when evaluating AF impacts and suggest that targeted emission control policies focusing on fossil-fuel and combustion sources may yield substantial cardiovascular benefits.

## Supplementary Material



## Notes

Additional methodological details, supplementary tables, and sensitivity analyses (PDF)
