# Supplementary material for: Source-Specific PM2.5 Exposure and Atrial Fibrillation Risk in U.S. Older Adults: A Nationwide Cohort Study
Source: Environ Health (Wash). 2026 Jun 22;4(10):2301–11. doi: 10.1021/envhealth.5c00638 (PMC13681434; doi:10.1021/envhealth.5c00638)
Supplement: Supplementary file 1 [file eh5c00638_si_001.pdf]

Supporting Information

Source-Specific $PM_{2.5}$ Exposure and Atrial Fibrillation Risk in U.S. Older Adults: A Nationwide Cohort Study

Qiao Zhu [a], Hua Hao [a], Yanling Deng [a], Yan V. Sun [b,c,d], Chang Liu [b], Alvaro Alonso [b], Yang Liu [a,*]

[a] Gangarosa Department of Environmental Health, Rollins School of Public Health, Emory University, Atlanta, GA 30322, USA

[b] Department of Epidemiology, Rollins School of Public Health, Emory University, Atlanta, GA 30322, USA

[c] Division of Cardiology, Department of Medicine, Emory University School of Medicine, Emory University, Atlanta, GA 30322

[d] Atlanta Veterans Affairs Health Care System, Decatur, GA 30033, USA

*Address correspondence to Yang Liu (yang.liu@emory.edu)

Table of Contents

Table S1. ICD-codes for Atrial fibrillation (AF) used in Medicare Chronic Conditions Warehouse (CCW) Algorithms.

Table S2. Baseline characteristics of the study population and distribution of source-specific $PM_{2.5}$ exposures after the 5-year clean period.

Table S3. Continuous hazard ratios for atrial fibrillation by $PM_{2.5}$ source-specific exposure, using three-year versus five-year clean period.

Table S4. Continuous hazard ratios for atrial fibrillation by $PM_{2.5}$ source-specific exposure with three-year clean period: 25-km buffer and non-mover cohort analyses.

Table S5. Spearman correlation matrix among source-specific $PM_{2.5}$ components.

Figure S1 Spatial distribution of $PM_{2.5}$ monitoring stations and 100-km buffer zones. (a) Locations of 515 $PM_{2.5}$ monitoring stations from the CSN and IMPROVE networks across the contiguous U.S. (red points). (b) The same stations with 100-km buffer zones (blue circles); only ZIP codes with centroids inside these zones were included.

Figure S2 Map of geographical regions (West, Midwest, Southwest, Southeast, Northeast) in the contiguous United States.

Table S1. ICD-codes for Atrial fibrillation (AF) used in Medicare Chronic Conditions Warehouse (CCW) Algorithms.

Algorithms	ICD-9	ICD-10	Number/Type of claims to qualify
**AF**	427.31 (ONLY first or second DX on the claim)	I48.0, I48.1, I48.11, I48.19, I48.2, I48.20, I48.21, I48.91 (ONLY first or second DX on the claim)	At least 1 inpatient claim OR 2 HOP/Carrier claims with DX codes

Note: DX denotes diagnosis; ICD-10 codes are effective 10/2015.

Table S2. Baseline characteristics of the study population and distribution of source-specific $PM_{2.5}$ exposures after the 5-year clean period.

**Characteristics**	**N = 20,598,240**
Number of AF events	3,429,283 (16.6)
Total person-years	171,555,756
Median follow-up years	5 (7)
Age at entry (years)	75.77 (6.35)
Age < 75 years	11,903,430 (57.8)
Age ≥ 75 years	8,694,810 (42.2)
**Sex**	
Male	8,254,581 (40.1)
Female	12,343,659 (59.9)
**Race**	
White	18,011,289 (87.4)
Black	1,603,507 (7.8)
Other [a]	983,444 (4.8)
**Medicaid eligibility**	
Ineligible	17,015,722 (82.6)
Ever eligible	3,582,518 (17.4)
**$PM_{2.5}$ sources ($\mu g/m^3$) [b]**	
Soil and dust	0.23 (0.14)
Vehicle emission	1.35 (0.55)
Biomass burning	1.01 (0.55)
Secondary Sulfate	2.21 (1.69)
Secondary Nitrate	1.11 (1.14)
Heavy oil and coal combustion	0.89 (0.91)
SOA	0.61 (0.76)

Data are summarized as mean (standard deviation) for continuous variables and count (percentage) for categorical variables, unless otherwise specified.

[a] Other included Asian, Hispanic, American Indian, or Alaskan Native, and unknown.

[b] Presented as median concentration (IQR).

Abbreviations: SD, standard deviation; IQR: interquartile range.

Table S3. Continuous hazard ratios for atrial fibrillation by $PM_{2.5}$ source-specific exposure, using three-year versus five-year clean period.

Exposure	3-year clean period	5-year clean period
**Soil**	1.006 (1.005, 1.008)	1.009 (1.007, 1.01)
**Vehicle emission**	1.029 (1.028, 1.03)	1.026 (1.025, 1.028)
**Biomass burning**	1.002 (1.001, 1.004)	0.997 (0.995, 0.999)
**Secondary Sulfate**	1.056 (1.053, 1.058)	1.062 (1.059, 1.064)
**Secondary Nitrate**	1.032 (1.03, 1.034)	1.033 (1.031, 1.036)
**Heavy oil and coal combustion**	1.048 (1.046, 1.05)	1.053 (1.051, 1.056)
**SOA**	0.985 (0.983, 0.987)	0.978 (0.975, 0.98)

Note: Models adjusted for temperature, relative humidity, smoking rate, BMI, hospital availability, and ZIP code–level socioeconomic covariates (population density, racial composition, median household income, renting rate, poverty, and education level), in addition to calendar year and geographic region of the United States.

Table S4. Continuous hazard ratios for atrial fibrillation by $PM_{2.5}$ source-specific exposure with three-year clean period: 25-km buffer and non-mover cohort analyses.

Exposure	25-km buffer	non-mover cohort
**Soil**	1.002 (1, 1.004)	1.006 (1.004, 1.007)
**Vehicle emission**	1.007 (1.006, 1.009)	1.025 (1.024, 1.027)
**Biomass burning**	0.983 (0.98, 0.986)	1.003 (1.002, 1.005)
**Secondary Sulfate**	1.06 (1.055, 1.064)	1.037 (1.034, 1.04)
**Secondary Nitrate**	1.01 (1.007, 1.013)	1.025 (1.023, 1.028)
**Heavy oil and coal combustion**	1.052 (1.048, 1.056)	1.047 (1.045, 1.05)
**SOA**	0.974 (0.972, 0.977)	0.976 (0.974, 0.978)

Note: Models adjusted for temperature, relative humidity, smoking rate, BMI, hospital availability, and ZIP code–level socioeconomic covariates (population density, racial composition, median household income, renting rate, poverty, and education level), in addition to calendar year and geographic region of the United States.

Table S5. Spearman correlation matrix among source-specific $PM_{2.5}$ components.

exposure	Soil	Vehicle emission	Biomass burning	Secondary Sulfate	Secondary Nitrate	Heavy oil and coal combustion	SOA
**Soil**	1.00	0.18	0.49	0.02	0.09	-0.21	0.38
**Vehicle emission**	0.18	1.00	0.29	0.16	0.67	0.31	0.11
**Biomass burning**	0.49	0.29	1.00	-0.05	0.13	-0.42	0.50
**Secondary Sulfate**	0.02	0.16	-0.05	1.00	0.46	0.61	0.17
**Secondary Nitrate**	0.09	0.67	0.13	0.46	1.00	0.32	-0.05
**Heavy oil and coal combustion**	-0.21	0.31	-0.42	0.61	0.32	1.00	-0.18
**SOA**	0.38	0.11	0.50	0.17	-0.05	-0.18	1.00

(a)

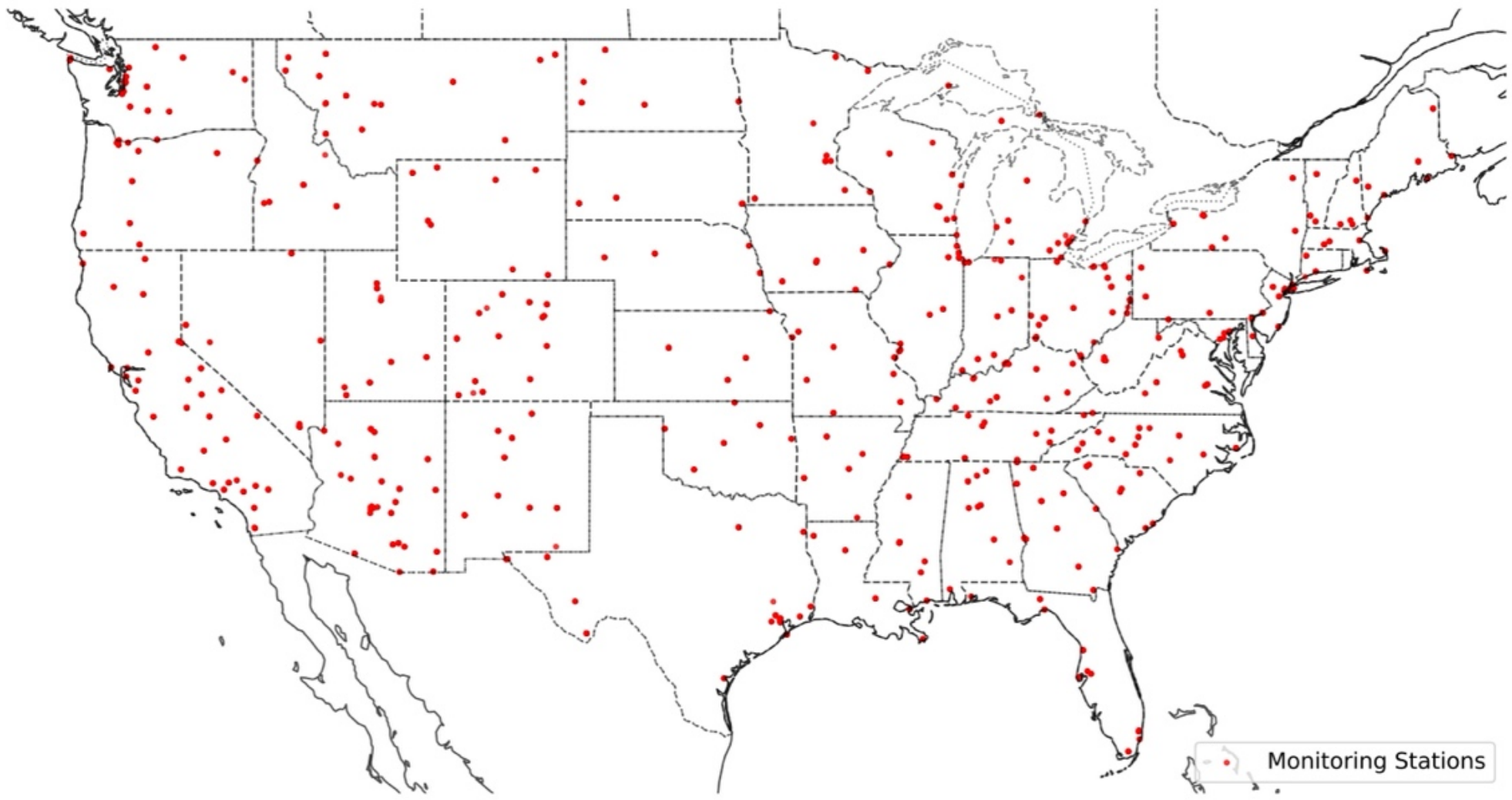

(b)

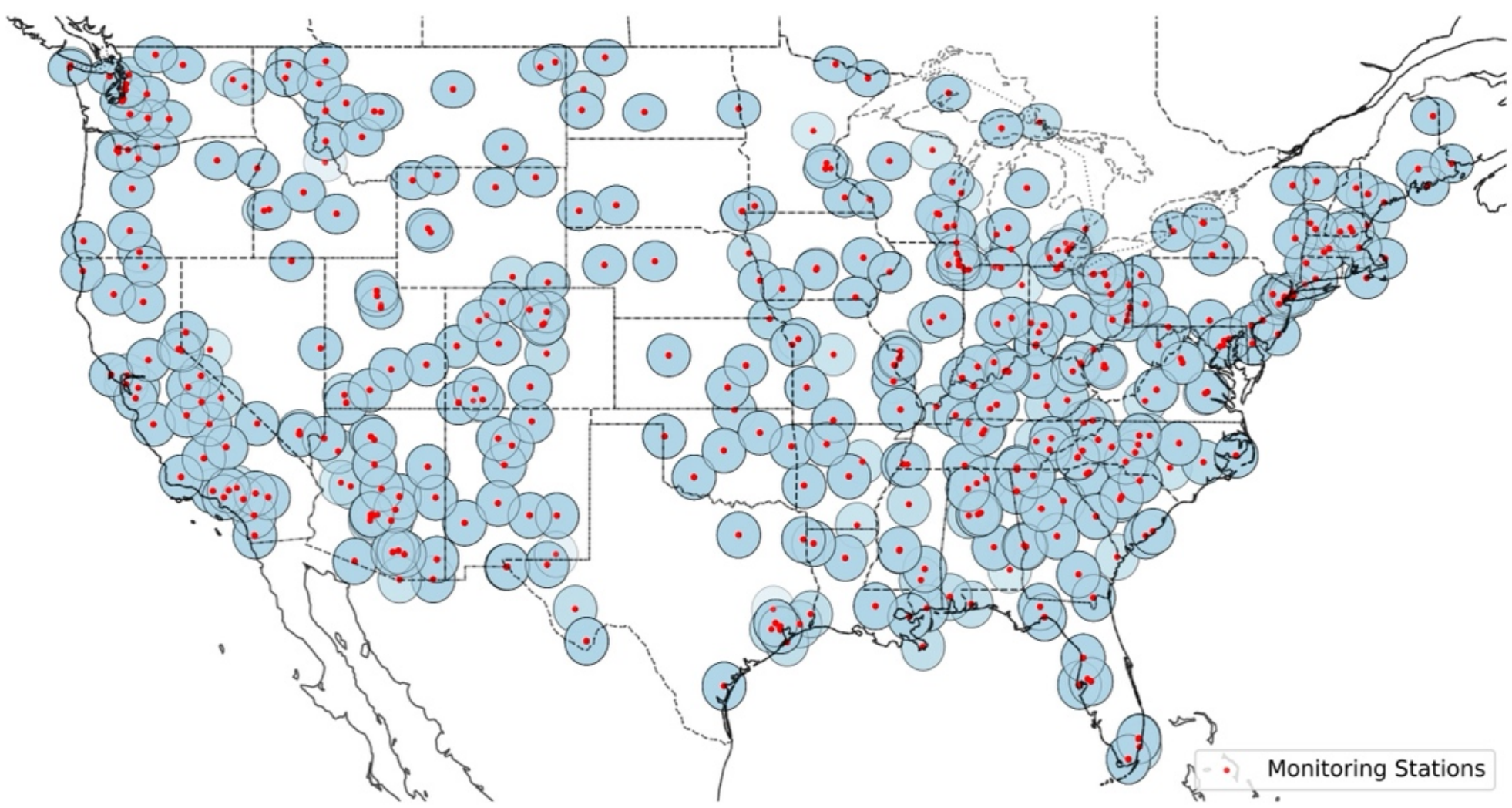

Figure S1 Spatial distribution of $PM_{2.5}$ monitoring stations and 100-km buffer zones. (a) Locations of 515 $PM_{2.5}$ monitoring stations from the CSN and IMPROVE networks across the contiguous U.S. (red points). (b) The same stations with 100-km buffer zones (blue circles); only ZIP codes with centroids inside these zones were included.

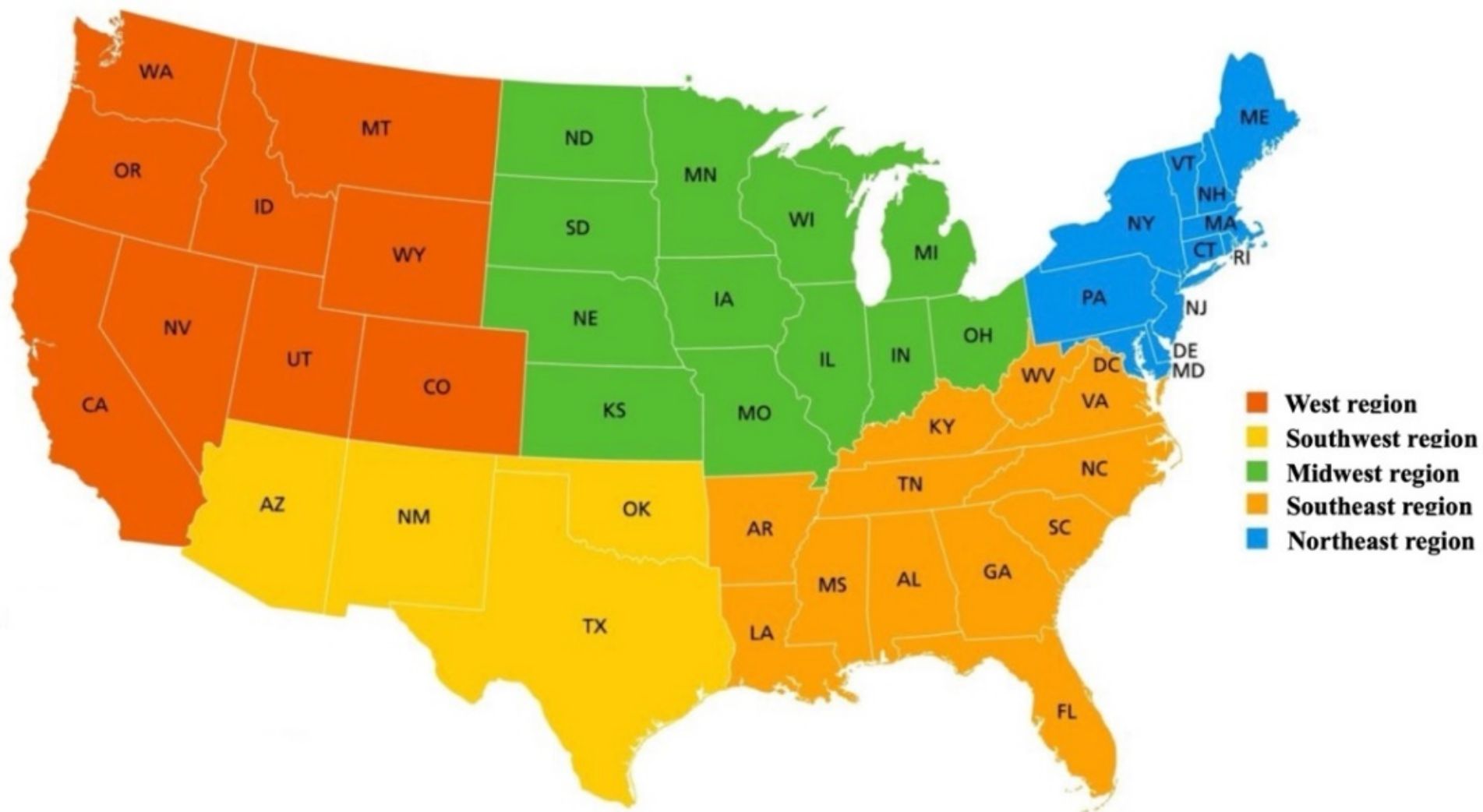

Figure S2 Map of geographical regions (West, Midwest, Southwest, Southeast, Northeast) in the contiguous United States.
